# A Novel Role of Pipecolic Acid Biosynthetic Pathway in Drought Tolerance through the Antioxidant System in Tomato

**DOI:** 10.3390/antiox10121923

**Published:** 2021-11-30

**Authors:** Ping Wang, Qian Luo, Weicheng Yang, Golam Jalal Ahammed, Shuting Ding, Xingyu Chen, Jiao Wang, Xiaojian Xia, Kai Shi

**Affiliations:** 1 Department of Horticulture, Zhejiang University, Hangzhou 310058, China; 11916061@zju.edu.cn (P.W.); 11916062@zju.edu.cn (Q.L.); 3170100209@zju.edu.cn (W.Y.); ahammed@haust.edu.cn (G.J.A.); 3130100256@zju.edu.cn (S.D.); 12016053@zju.edu.cn (X.C.); 11616046@zju.edu.cn (J.W.); xiaojianxia@zju.edu.cn (X.X.); 2 Zhejiang Provincial Key Laboratory of Horticultural Plant Integrative Biology, Hangzhou 310058, China

**Keywords:** pipecolic acid, CRISPR-Cas9, drought resistance, photosystems, antioxidants

## Abstract

With global warming and water shortage, drought stress is provoking an increasing impact on plant growth, development, and crop productivity worldwide. Pipecolic acid (Pip) is an emerging lysine catabolite in plants, acting as a critical element in disease resistance with a related signal pathway of phytohormone salicylic acid (SA). While SA plays a vital role in various abiotic stresses, the role of Pip in plant response to abiotic stresses, especially drought, remains largely unknown. To address this issue, Pip biosynthetic gene *Slald1* mutants and hydroxylated modification gene *Slfmo1* mutants were generated using CRISPR-Cas9 gene-editing approaches. Drought resistance dramatically increased in *Slald1* mutants compared with wild-type, which was associated with increased CO_2_ assimilation, photosystems activities, antioxidant enzymes activities, ascorbate and glutathione content, and reduced reactive oxygen species accumulation, lipid peroxidation and protein oxidation. On the contrary, *Slfmo1* mutants were more sensitive to drought, showing damaged photosystems and impaired antioxidant systems, which were significantly alleviated by exogenous ascorbate. Our results demonstrate that Pip biosynthesis and hydroxylated modification pathways play a critical role in drought tolerance through the antioxidant system in tomato. This knowledge can be helpful to breed improved crop cultivars that are better equipped with drought resistance.

## 1. Introduction

With global warming, rainfall disparity, and poor drainage, freshwater resources are becoming increasingly scarce [1,2]. Drought is one of the severe environmental handicaps for sustainable agriculture development [3]. Drought-related crop production losses reached over $30 billion worldwide during the last decade [4]. Drought stress alters various morphological, biochemical, and physiological features in plants [5,6]. With regards to morphological changes, reduced leaf area and restricted stem elongation are typical outcomes of the drought. Physiological and biochemical changes include turgor loss, photosynthetic inhibition, cytoplasmic membrane damages, and excessive reactive oxygen species (ROS) accumulation.

In the initial period of drought stress, stomatal closure reduces water loss via transpiration. A decreased CO_2_ availability inside the leaves leads to reduced energy consumption through the Calvin-Benson cycle [7,8]. At the cellular level, drought signals trigger the production of ROS, such as H_2_O_2_, superoxide anion radical (O_2_^**·**−^), and singlet oxygen, which may cause oxidative burst [9]. The photosystem II (PSII) and PSI are known to be the major sites of ROS generation [10,11]. Nevertheless, excessive ROS cause oxidative stress, impair photosynthetic machinery, and are toxic to cells [12]. Plants activate the antioxidant systems that deploy antioxidant enzymes including peroxidase enzymes to prevent acute cell damage and maintain membrane integrity and redox homeostasis [4].

Pipecolic acid (Pip) is a lysine-derived non-protein heterocyclic amino acid commonly found in various organisms, including bacteria, fungi, animals, and plants [13,14,15,16]. In *Arabidopsis*, Pip is biosynthesized from L-Lys by the aminotransferase AGD2-like defense response protein (ALD1) [17]. Flavin-dependent monooxygenase (FMO1) acts as a pipecolate N-hydroxylase, catalyzing hydroxylated modification of Pip to N-hydroxypipecolic acid (NHP) [18]. This biosynthetic pathway is also conserved in tomato (*Solanum lycopersium*) [19]. Pip is a critical regulator of inducible plant immunity because of its function in the activation of systemic acquired resistance (SAR) in response to pathogen attack [18]. Furthermore, Pip preconditions plants for optimal production of the phenolic defensive signal salicylic acid (SA) and orchestrates SAR and defense priming by both SA-dependent and SA-independent signaling mechanisms, playing a major and a minor role, respectively [20,21]. Ubiquitous hormone SA plays multiple roles in various abiotic stresses, whereas the roles of Pip in abiotic stresses, especially drought stress, remain largely unknown [22,23,24]. Expression profiling datasets of the genes from public databases show that *ALD1* and *FMO1* levels are significantly changed by drought stress [25]. Therefore, Pip seems to have a role in plant drought tolerance, which needs further investigation.

To survive under adverse conditions, plants have developed a diverse range of protective mechanisms, including cyclic electron flow (CEF) around photosystem I (PSI), a repair cycle for damaged photosystem II (PSII) reaction centers, and antioxidant pathways [5,26,27]. Previous research demonstrated that Pip induced resistance against pathogens in tomato, possibly through the regulation of ROS accumulation [28]. In addition, SA, which participates in the signal transduction pathway related to Pip, exerts resistance to abiotic stress by affecting the photosystems and antioxidant system of plants [29,30]. However, the role of Pip in drought resistance and its relationship with the photosystems and antioxidant system in plants, warrants more investigation.

Tomato (*S. lycopersicum* L.) is a nutritious fruit vegetable among the most widely grown crops in the world. However, many tomato genotypes are relatively tall with continuous flowering and fruiting habits, which make them generally sensitive to drought stress, causing tremendous yearly losses in tomato yield [31]. Therefore, developing tomato plants with improved water use efficiency is essential to minimize drought-induced losses of yield [32]. In this study, *Slald1* and *Slfmo1* mutants were generated to examine the role of Pip in drought stress. Results showed that *Slald1* mutants are relatively resistant, while *Slfmo1* mutants are more sensitive to drought. These effects are closely linked to respective changes in the photosystems and antioxidant system. Our results demonstrate the role of Pip biosynthetic and hydroxylated modification pathways in drought tolerance in tomato, which is potentially helpful to develop drought-resistant germplasms.

## 2. Materials and Methods

### 2.1. Plant Material, Growth Condition, and Drought Treatment

The tomato (*S. lycopersicum* L.) variety Condine Red (CR) from TGRC (Tomato Genetics Resource Center), UC DAVIS was used as the wild-type (WT) in the present study. Seeds were germinated in the vermiculite and perlite (*v*/*v* = 1:1) containing growth substrates. Following emergence, seedlings were moved in groups of four to 1.5 L tanks filled with Hoagland’s nutrient solution. An electric air pump was used to constantly aerate the solution. Tomato plants were cultivated under the following conditions in controlled growth chambers: 400 µmol m^−2^ s^−1^ photosynthetic photon flux density (PPFD), 14 h/10 h (day/night) photoperiod, 25 °C/20 °C (day/night) air temperature, and 75% relative humidity. Drought treatment was performed on around 5-week-old plants, and 5% (*w*/*v*) polyethylene glycol (PEG), with an average molecular weight of 6000 (PEG6000, Sigma-Aldrich, St. Louis, MO, USA), was used to simulate drought stress. As a control, a nutrient solution lacking PEG was provided. Two days after the drought stress treatment, chlorophyll fluorescence parameters, P700 absorbance, and ROS-related parameters were measured, as described below. For L-ascorbic acid (AsA, Hushi, Shanghai, China) pretreatment assay, each plant was sprayed with 20 mL 10 mM AsA or H_2_O as control once per day at night for three consecutive days before drought treatment. For pipecolic acid (Pip, Sigma-Aldrich, St. Louis, MO, USA) pretreatment assay, plants were fed with Hoagland’s nutrient solution with 1 mM Pip or without as control for two days before drought treatment according to Navarova et al. [33].

### 2.2. Construction of Plant Expression Vector and Tomato Transformation

National Center for Biotechnology Information (NCBI) protein Basic Local Alignment Search Tool (BLAST) (https://blast.ncbi.nlm.nih.gov/Blast.cgi (10 March 2019) was used to compare the percent amino acid identity between the two Pip-related genes *ALD1* and *FMO1* in *Arabidopsis* and their closest homologs in tomato, and named them as *SlALD1* and *SlFMO1*, respectively. CRISPR/Cas9 gene-editing-mediated *Slald1* and *Slfmo1* mutants in the cv CR background were generated according to Hu et al. [34]. The CRISPR/Cas9 gene-editing vector construction and the homozygous mutant plants were verified using PCR assays (primer sequences are given in Supplementary Table S1).

### 2.3. qRT-PCR Assay

Total RNA was extracted from plant tissues using the Trizol reagent (Easy-Do, Zhejiang, China) and used for reverse transcription reactions (Toyobo, Tokyo, Japan). Quantitative real-time PCR (qRT-PCR) was performed on optical 96-well plates in the Roche Light Cycler 480 instrument using SYBR SuperMix (Vazyme Biotech, Nanjing, China). *SlACTIN* was used as the internal standard, and the relative gene expression was calculated according to the 2^−ΔΔCT^ method. Supplementary Table S1 lists the primers used for the target genes and internal control *ACTIN* gene.

### 2.4. Subcellular Localization

*SlALD1* and *SlFMO1* were cloned into vectors with a GFP tag at the C-terminus under the control of the 35S CaMV promoter. The constructed vectors were transformed into *Agrobacterium tumefaciens* strain GV3101, and then transiently overexpressed in tobacco leaves, which contained a nuclear localization protein that could emit a red fluorescent signal (NLS-mCherry). Confocal laser observation (Zeiss LSM 780, Oberkochen, Germany) was performed two days later. The GFP signal was detected at excitation wavelengths of 488 nm and emission between 500 nm and 530 nm. The excitation of NLS-mCherry was conducted at 561 nm, with emissions being captured between 580 and 620 nm. The autofluorescence chloroplast emission spectrum was between 650 and 720 nm. Supplementary Table S1 lists relevant primers used in the experiments.

### 2.5. Leaf Gas Exchange Measurements

A portable photosynthesis system (LI-6400T, Li-Cor Inc., Lincoln, NE, USA) was used to measure net photosynthetic rate (*P*n), intercellular CO_2_ concentration (*C*i), stomatal conductance (*G*s), and transpiration rate (*T*r), which were recorded when photosynthesis reached a steady state. The detection conditions were similar to the growth conditions: 400 µmol m^−2^ s^−1^ photosynthetic photon flux density (PPFD), approximately 400 ppm atmospheric CO_2_ concentrations, and 25 °C leaf temperature.

### 2.6. Chlorophyll Fluorescence Measurements

Chlorophyll fluorescence parameters were measured with the MAXI Version of the Imaging-PAM M-Series chlorophyll fluorescence system (Heinz-Walz, Effeltrich, Germany). Plants were adapted under dark for 30 min before measurement. The initial fluorescence (Fo), maximum fluorescence yield in the dark (Fm), and the maximum fluorescence yield under light-adapted state (Fm′) was determined according to Jiang et al. [35]. The chlorophyll fluorescence parameters were calculated as follows: effective quantum yield of PSII, Y(II) = (Fm′ − F)/Fm′; quantum yield of non-regulatory energy dissipation, Y(NO) = F/Fm; maximum photochemical quantum yield of PSII, Fv/Fm = (Fm − Fo)/Fm; non-photochemical quenching in PSII, NPQ = (Fm − Fm′)/Fm′; photochemical quenching, qP = (Fm′ − F)/(Fm′ − Fo′). qE was simultaneously measured with the Dual-PAM-100 system (Heinz-Walz, Effeltrich, Germany) according to Jiang et al. [35] and calculated according to the equations qE = Fm/Fm′ − Fm/Fm″ [36].

The ΔP700_max_ (Pm) was determined using a saturation pulse under an FR background according to Klughammer et al. [37] The decrease in Pm is an indicator of PSI photoinhibition. The capacity of CEF around PSI was determined by the half time of dark rereduction of P700^+^ (t_1/2_) signal after switching off the FR light [38]. A quantitative assay of CEF via the postillumination rereduction of P700^+^ was based on Jiang et al. [35]. Photochemistry quantum yield of PSI photochemistry, Y(I) = (Pm′ − P)/Pm; quantum yield of non-photochemical energy dissipation owing to acceptor side limitation, Y(NA) = (Pm − Pm′)/Pm [27].

### 2.7. Detection of Lipid Peroxidation and Electrolyte Leakage

The degree of lipid peroxidation was evaluated by measuring the quantity of MDA generated by the thiobarbituric acid reaction according to Hodges et al. [39]. Membrane permeability was assessed using a technique published by Cao et al. [40] after exposure of tomato seedlings to drought.

### 2.8. ROS Analysis

To assess ROS accumulation, leaves were stained with DAB and NBT to detect H_2_O_2_ and O_2_^**·**−^ accumulation in situ as previously explained [34]. The concentrations of H_2_O_2_ in the leaves were measured according to the method described previously by recording the changes in absorbance at 412 nm [41] with minor modifications [42]. The O_2_^**·**−^ accumulation was detected with an O_2_^**·**−^ Detection Kit (sulfonamide color-based method) (Yuanye Biology, Shanghai, China) following the manufacturer’s manuals. The O_2_^**·**−^ content was determined by monitoring the absorbance at 530 nm.

### 2.9. Immunoblotting Assay

The oxidized protein fractions obtained from the soluble protein were analyzed using an OxyBlot Protein Oxidation Detection Kit (Chemicon International, Temecula, CA, USA) in accordance with the manufacturer’s instructions.

### 2.10. Antioxidant Content and Enzyme Activity Assays 

For the non-enzymatic antioxidants, such as ascorbate and glutathione assays, about 100 mg leaf tissues were ground to a fine powder in liquid nitrogen and extracted into 1 mL 0.2 M HCl. The following sample neutralizes enzyme preparation, detection, and calculation according to Noctor et al. [43].

For antioxidant enzyme activity assays, 300 mg leaf tissues were ground with 3 mL ice-cold enzyme buffer containing 25 mM HEPES, 0.2 mM EDTA, 2 mM AsA, and 2% polyvinylpolypyrrolidone (*w*/*v*) (pH 7.8). SHIMADZU UV-2410PC spectrophotometer (Shimadzu, Kyoto, Japan) was used to detect the subsequent enzyme activity. The enzyme activities of APX, DHAR, CAT, and GR were analyzed according to Hu et al. [42]. The enzyme activities of SOD and POD were measured following the previously described protocol [44].

### 2.11. Statistical Analysis

The experiments were performed under a completely randomized design with three replications. Each replication had a minimum of 12 plants. The differences among treatment means were determined via SAS statistical package, followed by Tukey’s test at *p* < 0.05.

## 3. Results

### 3.1. Changes in the Transcript Levels of Tomato Pip Biosynthetic and Hydroxylated Modification Genes in Response to Drought Stress

**Figure 1:**
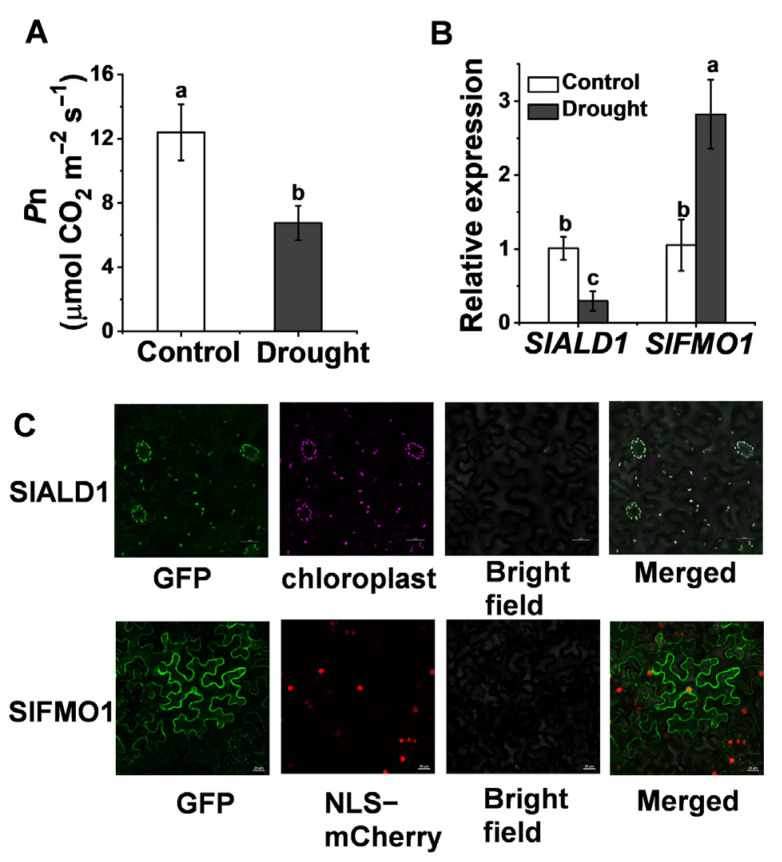
Changes in the transcript levels of tomato pip biosynthetic and hydroxylated modification genes in response to drought stress. (**A**) The net carbon dioxide (CO_2_) assimilation rate, *P*n, in wild-type (WT) plants at 48 h after control or drought treatment. (**B**) Relative expression of *SlALD1* and *SlFMO1* in WT plants leaves at 24 h after control or drought treatment. (**C**) Subcellular localization of SlALD1 and SlFMO1. The tomato SlALD1-GFP and SlFMO1-GFP plasmids were transiently expressed in *N. benthamiana* leaves. Through confocal microscopy, the GFP, autofluorescence chloroplast, and NLS-mCherry (a marker for nuclear localization) signals were visualized at 48 h after infiltration. Bar = 25 µm. Different letters (a, b, c) above each bar (*n* = 4) represent significant differences (*p* < 0.05).

Based on the similarity to the amino acid sequences of ALD1 (AT2G13810) and FMO1 (AT1G19250) in *Arabidopsis*, we identified tomato Pip biosynthetic gene *SlALD1* (Solyc11g044840) and hydroxylated modification gene *SlFMO1* (Solyc07g04243) in the tomato genome. SlALD1 and SlFMO1 showed 63.82% and 65.04% similarity to ALD1 and FMO1, respectively. Drought stress injures the photosynthetic system of plants, resulting in a significant decrease in the net CO_2_ assimilation rate, *P*n (Figure 1A). To investigate the response of tomato Pip biosynthesis and modification genes to drought stress, qRT-PCR was used to analyze the expression of *SlALD1* and *SlFMO1* under drought stress. The expression of *SlALD1* reduced by 29.2% under drought stress, whereas the expression of *SlFMO1* increased 2.7 times compared to control (Figure 1B). Expression profiles of *ALD1* and *FMO1* in *Arabidopsis* subjected to drought stress were retrieved from public databases [25], which showed a similar trend to tomato. To determine the subcellular localization of SlALD1 and SlFMO1, 35S: SlALD1-GFP and 35S: SlFMO1-GFP constructs were transiently expressed in *Nicotiana benthamiana* leaves. It was observed that SlALD1-GFP localized at the chloroplast, whereas SlFMO1-GFP localized at the plasma membrane and cytoplasm (Figure 1C).

### 3.2. Effects of Drought Stress on Slald1 and Slfmo1 Mutants

**Figure 2:**
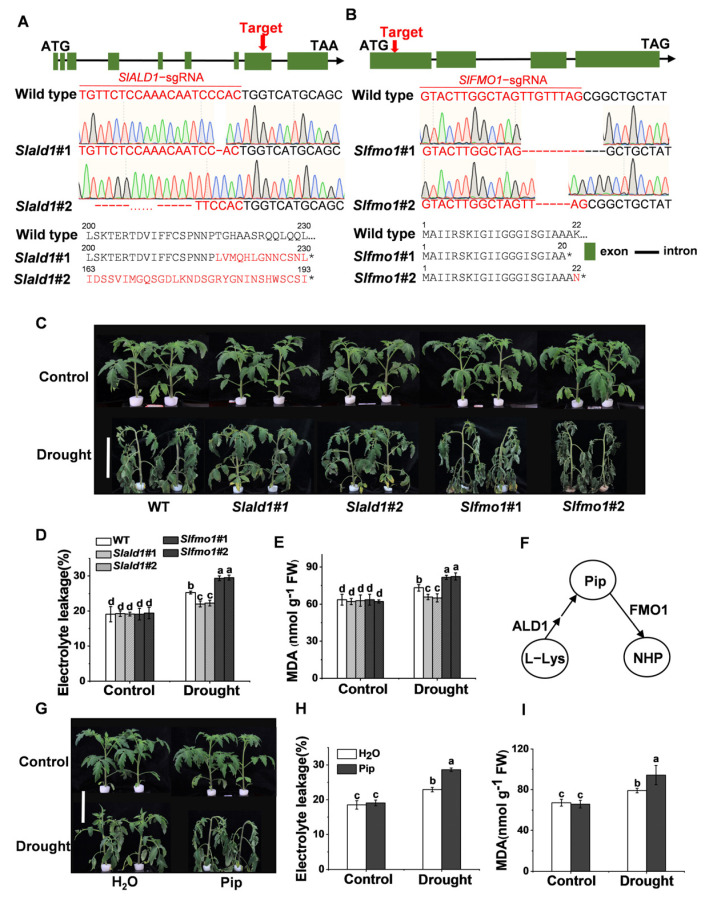
Effects of drought stress on *Slald1* and *Slfmo1* mutants. (**A**) Schematic illustration of the sgRNA target site (red arrows), DNA sequencing peak map, and protein sequence in wild-type (WT) *SlALD1* and two alleles (*Slald1*#1 and *Slald1*#2) from CRISPR-Cas9 T2 mutant lines. (**B**) Schematic illustration of *Slfmo1*#1 and *Slfmo1*#2. (**C**) Representative images of *Slald1*, *Slfmo1* mutants and WT plants. Bar = 10 cm. The mutants and WT plants were subjected to control or drought treatment, and the plant images were taken 48 h later. (**D**) The relative electrolyte leakage of tomato leaves after 48 h of control and drought treatments. (**E**) The membrane lipid peroxidation product MDA accumulation in tomato leaves after 48 h of control and drought treatments. (**F**) Schematic illustration of Pip biosynthesis and downstream N-hydroxylated modification. (**G**) Representative images of plants pretreated with H_2_O and 1 mM Pip under control and drought treatments. Bar = 10 cm. Plant images were taken 48 h later. (**H**) The relative electrolyte leakage of tomato leaves after 48 h of control and drought treatments. (**I**) MDA content in tomato leaves after 48 h of control and drought treatments. The data are presented as mean values ± SD, *n* = 4. Different letters (a, b, c, d) above each bar indicate significant differences at *p* < 0.05 (Tukey’s test) among treatments.

To explore the function of SlALD1 and SlFMO1 in drought stress, CRISPR/Cas9-mediated gene editing technology was used to generate *Slald1* and *Slfmo1* mutants in tomato. Homozygous gene edited lines *Slald1*#1 and *Slald1*#2 (1bp and 91bp deletion in exon leading to an early stop codon, respectively) (Figure 2A) were isolated and used for experiments. Similarly, *Slfmo1*#1 and *Slfmo1*#2 (10bp and 4bp deletion in exon leading to an early stop codon, respectively) were also used for subsequent experiments (Figure 2B). Four-week-old *Slald1*, *Slfmo1* mutants and WT plants were exposed to drought for 48 h. *Slald1* mutants displayed strikingly greater resistance to drought stress, while *Slfmo1* showed sensitivity to drought (Figure 2C). Drought stress led to significantly increased electrolyte leakage and malondialdehyde (MDA) levels. Consistent with the phenotypes, *Slald1* mutants showed lower electrolyte leakage and MDA content than WT, however, *Slfmo1* mutants showed the highest levels of electrolyte leakage and MDA content (Figure 2D,E).

Previous studies have shown that *ALD1* is a biosynthetic gene of Pip, and FMO1 acts as a downstream pipecolate N-hydroxylase to modify Pip to NHP (Figure 2F). The pathway of Pip biosynthesis and modification is conserved in *S. lycopersium* [19]. Therefore, we next investigated the effects of exogenous application of Pip on plant drought resistance to confirm the responses of *Slald1* and *Slfmo1* mutants. Plants supplemented with 1 mM Pip showed sensitivity to drought (Figure 2G). Accordingly, electrolyte leakage and MDA content increased in Pip-pretreated plants compared with the control (Figure 2H,I).

Taken together, these results demonstrated that Pip played a negative role in drought tolerance in tomato. Consistent with this, Pip biosynthetic gene mutants *Slald1* showed resistance to drought, and its downstream modified gene mutants *Slfmo1* showed sensitivity.

### 3.3. Changes in Gas Exchange Parameters and PSI in Slald1 and Slfmo1 Mutants under Drought Stress

**Figure 3:**
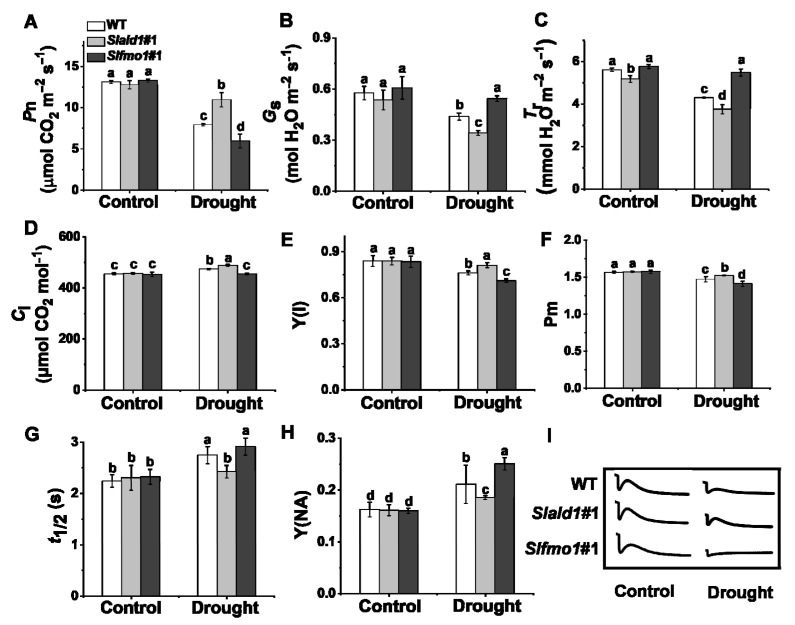
Changes in gas exchange parameters and PSI in *Slald1* and *Slfmo1* mutants under drought stress. The gas exchange parameters: net carbon dioxide (CO_2_) assimilation rate (**A**), stomatal conductance (**B**), transpiration rate (**C**), and intercellular CO_2_ concentration (**D**) of *Slald1*, *Slfmo1* mutants and WT plants after 48 h of control and drought treatment. PSI energy conversion: Y(I) (**E**), Pm (**F**), t_1/2_ (**G**), Y(NA) (**H**), and the cyclic electron flow (CEF) around PSI of *Slald1*, *Slfmo1* mutants and WT plants after 48 h of control and drought treatment (**I**). The data are presented as mean values ± SD, *n* = 4. Different letters (a, b, c, d) above each bar indicate significant differences at *p* < 0.05 (Tukey’s test) among treatments.

Photosynthesis is sensitive to drought stress due to prompt stomatal closure and attenuated electron transport [45]. The net CO_2_ assimilation rate, *P*n, of *Slald1* mutants was significantly higher than WT under drought stress, while *P*n of *Slfmo1* mutants was the lowest (Figure 3A). Due to the closed stomata, the leaf transpiration rate decreased, thereby reducing water loss. The stomatal conductance (*G*s) and transpiration rate (*T*r) of *Slald1* mutants were dramatically reduced by drought (Figure 3B,C). Nevertheless, the intercellular CO_2_ concentration (*C*i) of *Slald1* mutants was increased (Figure 3D). There were no differences in *G*s, *T*r and *C*i between WT and *Slfmo1* mutants under control conditions (Figure 3B–D).

Photosystem I (PSI) is a key source of ROS production, and it is also tightly connected to ROS-scavenging mechanisms in the chloroplast [10,46]. We next detected the changes in PSI under drought stress. We found that the quantum efficiency of PSI Y(I), and the maximum P700 photooxidation level Pm of WT plants were dramatically reduced by drought stress. *Slald1* deletion alleviated this reduction, while these were aggravated in *Slfmo1* mutants (Figure 3E,F). Besides, the half time of dark rereduction of P700^+^ (t_1/2_) extended, and the quantum yield of the acceptor side limitation of PSI Y(NA) significantly increased under drought in WT plants. *Slald1* mutations could alleviate this increase, while these were aggravated in *Slfmo1* mutants (Figure 3G,H). The cyclic electron flow (CEF) around PSI was inhibited by drought stress, while this inhibition state was attenuated in *Slald1* mutants, but more pronounced in *Slfmo1* mutants (Figure 3I). Nevertheless, *Slfmo1* mutants showed more damage to PSI under drought. These results indicate that *SlALD1* affects gas exchange, P700 oxidation, CEF and normal PSI status, thereby aggravating the drought-induced damages.

### 3.4. Changes in PSII in Slald1 and Slfmo1 Mutants under Drought Stress

**Figure 4:**
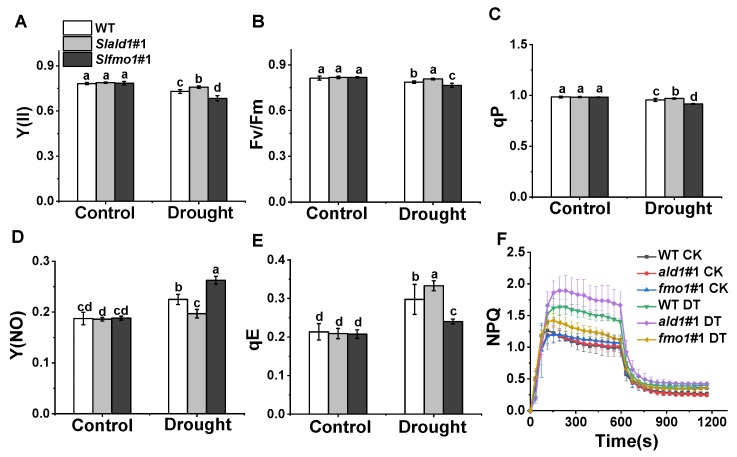
Changes in PSII in *Slald1* and *Slfmo1* mutants under drought stress. PSII energy conversion: Y(II) (**A**), Fv/Fm (**B**), qP (**C**), Y(NO) (**D**), qE (**E**) and NPQ kinetics (**F**) in *Slald1*, *Slfmo1* mutants and WT plants after 48 h of control and drought treatment. CK represents control, and DT represents drought. The data are presented as mean values ± SD, *n* = 4. Different letters (a, b, c, d) above each bar indicate significant differences at *p* < 0.05 (Tukey’s test) among treatments.

Drought stress causes damages to PSII photochemistry and induces photoinhibition [47]. Especially, rapid induction and release of non-photochemical quenching (NPQ) play crucial roles in protecting plants against photoinhibition [48]. We found that the effective quantum yield of PSII Y(II), the maximum photochemical efficiency of PSII, Fv/Fm, and the photochemical quenching, qP, reduced slightly under drought stress in WT plants, and these reductions hardly occurred in *Slald1* mutants, suggesting that *SlALD1* deletion could protect PSII from drought-induced damage (Figure 4A–C). However, these damages to PSII were more severe in *Slfmo1* mutants (Figure 4A–C). The quantum yield of non-regulatory energy dissipation Y(NO) in WT significantly increased under drought. This increase largely attenuated in *Slald1* mutants but aggravated in *Slfmo1* mutants (Figure 4D). Energy-dependent quenching qE is the main component of NPQ. Under drought stress, the qE and NPQ kinetics of WT plants increased to protect photosystem, and this increase was more pronounced in *Slald1* mutants (Figure 4E,F). Meanwhile, the increase was compromised in *Slfmo1* mutants (Figure 4E,F). These results suggest that deletion of *SlALD1* by gene editing approach could protect PSII from drought-caused damage and activate the NPQ system to protect plants. Nevertheless, *Slfmo1* mutants showed more damage to PSII under drought conditions.

### 3.5. Drought-Induced Changes in ROS Concentrations and Protein Oxidation in Slald1 and Slfmo1 Mutants

**Figure 5:**
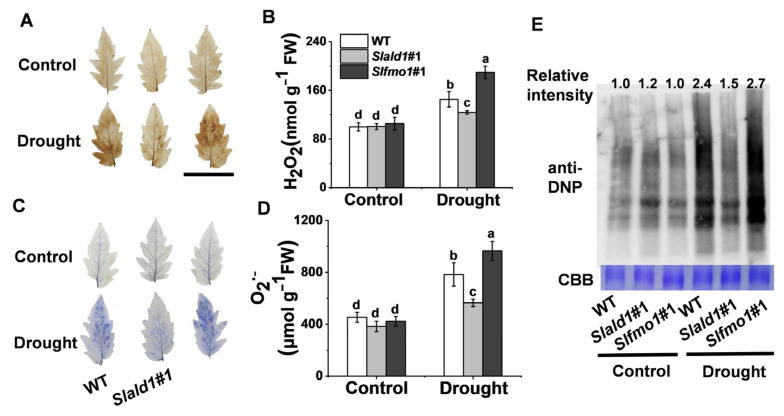
Drought-induced changes in ROS concentrations and protein oxidation in *Slald1* and *Slfmo1* mutants. (**A**) Representative images of in situ H_2_O_2_ accumulation detected by DAB staining. Bar = 5 cm (**B**) Quantification of H_2_O_2_. (**C**) Representative images of O_2_^**·**−^ accumulation as determined by NBT staining. (**D**) Quantification of O_2_^**·**−^. (**E**) Oxidized protein levels as detected by immunoblot analysis with anti-DNP antibody. Leaf samples were collected from *Slald1*, *Slfmo1* mutants and WT plants after 48 h of control and drought treatment. The data are presented as mean values ± SD, *n* = 4. Different letters (a, b, c, d) above each bar indicate significant differences at *p* < 0.05 (Tukey’s test) among treatments.

Drought stress typically causes oxidative stress through the accumulation of ROS [9]. Under control conditions, NBT and DAB staining and contents detection of O_2_^**·**−^ and H_2_O_2_ showed that the levels of O_2_^**·**−^ and H_2_O_2_ were not different in leaves between the mutants and WT. Under drought conditions, the concentrations of O_2_^**·**−^ and H_2_O_2_ decreased in *Slald1* mutants and increased in *Slfmo1* mutants compared with that in WT (Figure 5A–D). Proteins undergo structural changes as a result of drought, which has an impact on both protein quantity and turnover. We used 2,4-dinitrophenol (DNP) and anti-DNP antibodies to detect the carbonylation status of leaf proteins in *Slald1* and *Slfmo1* mutants and WT plants following drought stress in order to evaluate the impact of *SlALD1* and *SLFMO* on drought-dependent effects on protein characteristics. As shown in Figure 5E, SlALD1 deletion led to a lower drought-dependent elevation in the level of protein carbonylation, however, *Slfmo1* mutants showed a tremendous increase. Nevertheless, the levels of oxidized proteins in *Slald1*, *Slfmo1* mutants and WT plants were similar under control conditions. These findings suggest that the cellular redox homeostasis under drought was improved in the *Slald1* mutants, but damaged in the *Slfmo1* mutants.

### 3.6. Regulation of Cellular Redox Homeostasis in Slald1 and Slfmo1 Mutants under Drought

**Figure 6:**
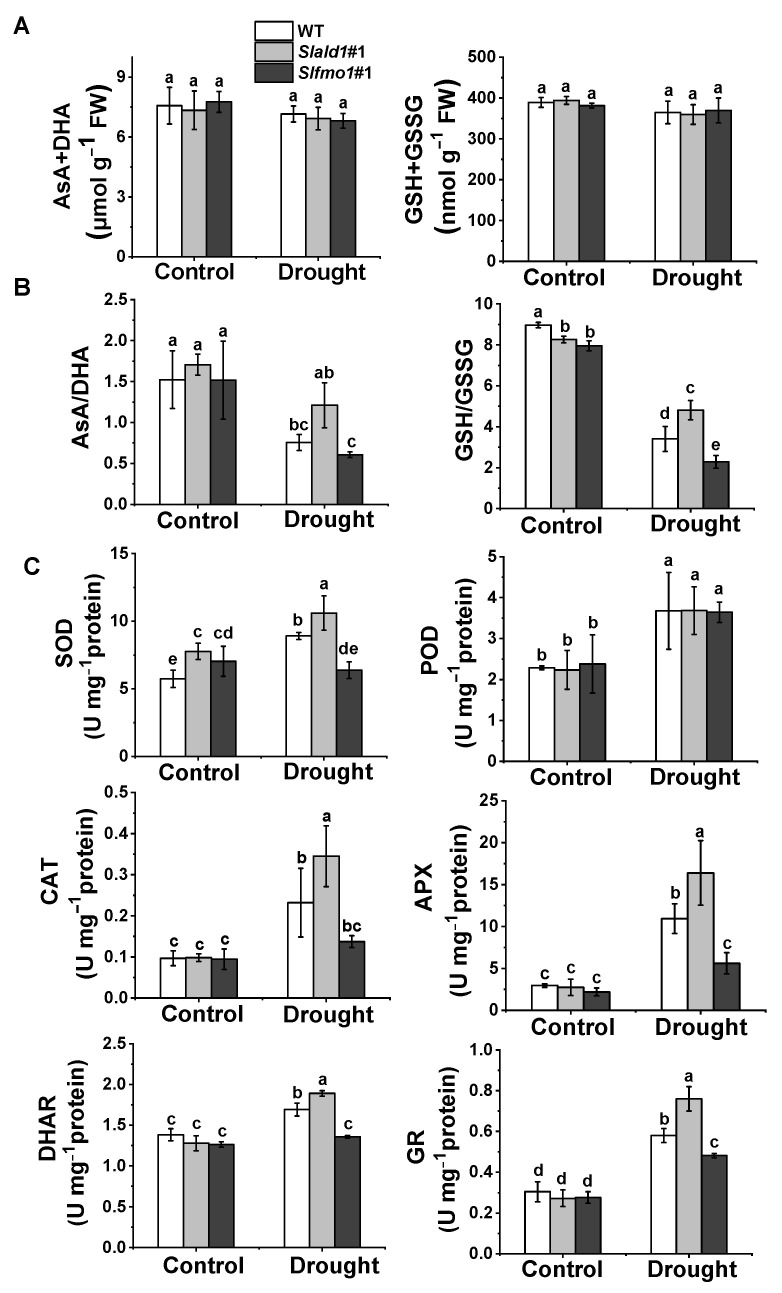
Regulation of cellular redox homeostasis in *Slald1* and *Slfmo1* mutants under drought. (**A**) Effects of drought treatment on total ascorbate (AsA + DHA) and glutathione (GSH + GSSG) contents, (**B**) Redox status, and (**C**) Antioxidant enzyme activities in mutants and WT plants. The *Slald1*, *Slfmo1* mutants and WT plants were subjected to control and drought treatment, and the leaves were sampled at 48 h after the initiation of treatment to analyze antioxidant content and enzyme activity. APX, ascorbate peroxidase; AsA, reduced ascorbate; CAT, catalase; DHA, dehydroascorbate; DHAR, dehydroascorbate reductase; GR, glutathione reductase; GSH, glutathione; GSSG, glutathione disulfide; POD, peroxidase; SOD, superoxide dismutase. The data are presented as mean values ± SD, *n* = 4. Different letters (a, b, c, d, e) above each bar indicate significant differences at *p* < 0.05 (Tukey’s test) among treatments.

Ascorbate and glutathione are ubiquitous and stable antioxidants that act as the heart of the cytosolic redox hub by maintaining adequate redox potentials in the cell. The redox state is indicated by shifts in the reduced ascorbate-to-dehydroascorbate (AsA/DHA) ratio and the reduced glutathione-to-glutathione disulfide (GSH/GSSG) ratio. There were no differences in the total levels of the ascorbate (AsA plus DHA) and glutathione (GSH plus GSSG) pools among the different lines, regardless of the water content (Figure 6A). However, AsA/DHA and GSH/GSSG ratios were higher in the *Slald1* mutants than in the WT plants under drought, while these ratios were the lowest in *Slfmo1* mutants (Figure 6B).

The activation of antioxidant enzymes such as superoxide dismutase (SOD), peroxidase (POD), ascorbate peroxidase (APX), catalase (CAT), dehydroascorbate reductase (DHAR), and glutathione reductase (GR) minimizes excessive ROS accumulation. In response to drought, the activity of all these antioxidant enzymes significantly elevated in *Slald1* mutants compared to the WT, except for POD (Figure 6C). Meanwhile, drought-induced such increases in the activity of antioxidant enzymes were compromised in *Slfmo1* mutants (Figure 6C). However, the levels of these antioxidant enzyme activities were not different between *Slald1*, *Slfmo1* mutants and WT plants under control conditions (Figure 6C).

These results suggest that deletion of *SlALD1* could increase the levels of non-enzymatic antioxidants and antioxidant enzymes under drought stress to relieve the damage caused by excessive ROS accumulation in plants, while *SlFMO1* played an opposite role.

### 3.7. Drought-Induced Sensitivity in Slfmo1 Mutants was Alleviated by Exogenous AsA

**Figure 7:**
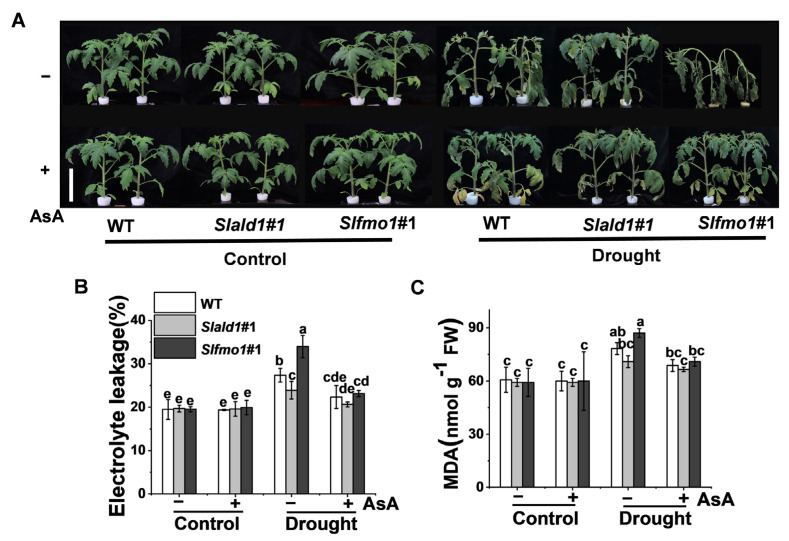
Drought-induced sensitivity in *Slfmo1* mutants was alleviated by exogenous AsA. (**A**) Phenotypes of tomato plants under control and drought conditions as influenced by AsA. Before exposure of the plants to control and drought treatment, 10 mM AsA or H_2_O (control) was applied on the foliage of the mutants and WT plants once per day for three consecutive days, and the images of plants were captured at 48 h after the drought treatment. Bar = 10 cm. (**B**) The relative electrolyte leakage in leaves after 48 h of control and drought treatment with or without AsA pretreatment. (**C**) Malondialdehyde (MDA) content in leaves after 48 h of control and drought treatments with or without AsA pretreatment. The data are presented as mean values ± SD, *n* = 4. Different letters (a, b, c, d, e) above each bar indicate significant differences at *p* < 0.05 (Tukey’s test) among treatments.

We then examined whether the drought-induced sensitivity in *Slfmo1* mutants could be alleviated by the exogenous application of AsA. As expected, the application of AsA increased drought resistance in all genotypes, including mutants and WT, and AsA could alleviate the drought-induced sensitivity in *Slfmo1* mutants to a similar level of WT plants (Figure 7A). Meanwhile, exogenous AsA could also decrease the electrolyte leakage and MDA content in *Slfmo1* mutants to a similar level compared with *Slald1* mutants and WT plants under drought (Figure 7B,C).

## 4. Discussion

Drought stress is a major abiotic factor that severely limits crop growth, development, and productivity [49]. It causes stomatal closure, photosystem injury, and ROS accumulation, thus exerting adverse effects on plants [50]. In response to these damaging effects, plants recruit antioxidant systems, including non-enzymatic antioxidant substances and antioxidant enzymes, to scavenge excess ROS, thereby increasing plant resistance to drought [51].

Higher plants have evolved multiple mechanisms to adapt to drought stress that involve plant defense hormones such as abscisic acid, SA, ethylene, as well as amino acids and derivatives [52]. Lysine metabolite Pip is related to SA in plant disease defense. Pip in plants exposed to all abiotic stresses changed significantly compared with the control [53], but little is known about its role in drought stress. In this study, the deletion of tomato Pip biosynthetic gene *SlALD1* showed drought resistance, and the hydroxylated modification gene *SlFMO1* deletion showed sensitivity to drought. Accordingly, Pip was found to aggravate drought sensitivity in tomato (Figure 2). In agreement with these results, *ALD1* and *FMO1* in *Arabidopsis* showed similar expression levels under drought stress [25]. In addition, there are other genes in Pip biosynthetic pathway, such as SAR-deficient 4 (*SARD4*), which can reduce 2,3-de-hydropipecolic acid (2,3-DP) to Pip, but its role in drought stress remains unknown. The downstream pathway of Pip in plant immunity also includes glucosylated NHP at the hydroxyl functional group to form NHP-O-glycoside (NHP-OGlc), and it is catalyzed by UGT76B1 [54]. Whether these signal metabolites and enzymes participate in the drought signal pathway of tomato plants remains to be explored.

Lysine is an essential amino acid derivated by distinct pathways. Lysine catabolism via saccharopine pathway (SACPTH) is highly responsive to abiotic stress but lacks evidence for biotic stress response [55]. Instead, previous studies showed that lysine metabolism through the NHP pathway seems preferentially associated with pathogen infection and may not contribute to abiotic stress response [25]. Here, a novel role of the pipecolic acid biosynthetic pathway in drought tolerance was found. It is the first study to investigate the role of lysine metabolism through the NHP pathway in tomato drought stress. Perhaps the biotic and abiotic stress responses associated with lysine catabolism through the SACPATH and the NHP pathway may not work independently as the previous hypothesis.

Drought stress generally entails ROS accumulation, photosystems injury, and changes in protein stability and turnover [42,51]. Drought stress is known to inhibit photosynthetic activity in tissues due to an imbalance between light capture and its utilization [56]. The maintenance of photosynthetic efficiency constitutes an essential mechanism of plant drought resistance [27]. Antioxidant enzymes are drought-responsive proteins that function against the negative impacts of drought stress [10]. Photosystems and antioxidant systems have an inseparable relationship. In our study, the deletion of *SlALD1* by gene-editing approach showed improved drought resistance, as reflected by a less impaired photosynthetic system and a more active antioxidant system. In contrast, the deletion of *SlFMO1* showed sensitivity to drought with a more impaired photosynthetic system and less active antioxidant system (Figure 2, Figure 3, Figure 4, Figure 5 and Figure 6). Furthermore, the exogenous application of AsA effectively alleviated the drought sensitivity in *Slfmo1* mutants (Figure 7). These results clearly demonstrate that the responses of Pip biosynthetic and downstream modified genes under drought are associated with photosystems and antioxidant systems. Notably, exogenous application of Pip induced resistance against *Pst* DC3000 and *B. cinerea* in tomatoes, also through the regulation of ROS accumulation and defense-related gene expression [28]. NHP pretreatment in wheat seedling alters the expression of genes related to cellular redox homeostasis [57]. Likewise, Pip is associated with peroxisomal-related disorders in mammals by modulating the activities of CAT, SOD, and other antioxidant enzymes [58]. Overall, Pip seems to be a critical metabolite that modulates the antioxidant system to mediate plant responses to biotic and abiotic stresses. Since both the SA-related signal pathway and Pip play roles in pathogen defense, the use of the SA signal mutants can clarify the relationship between Pip and SA in drought resistance, warranting further research in the future.

In summary, the data presented here show that the Pip biosynthetic and hydroxylated modification pathways play critical roles in drought stress response, through the modulation of cellular redox homeostasis. Moreover, deletion of *SlALD1* by CRISPR-Cas9-mediated gene-editing may be helpful to breed improved cultivars that are better equipped for drought resistance. Nevertheless, the role of endogenous Pip content in plant drought resistance is not well understood, and the effects of *SlALD1* deletion on fruit yield and quality under drought stress should be addressed in future studies.

## 5. Conclusions

This study reveals that Pip biosynthetic and hydroxylated modification pathways play critical roles in drought stress response by modulating cellular redox homeostasis. It is the first study to investigate the role of lysine metabolism through the NHP pathway in tomato drought stress. Moreover, deletion of *SlALD1* by CRISPR-Cas9-mediated gene-editing may be helpful to breed improved cultivars that are better equipped for drought resistance.

## Data Availability

The data supporting the findings of this study are available within the article and its Supplementary Materials.

## Supplementary Materials

The following are available online at https://www.mdpi.com/article/10.3390/antiox10121923/s1, Table S1: Primers used for various applications in the present study.
