# Supplementary material for: A Novel Role of Pipecolic Acid Biosynthetic Pathway in Drought Tolerance through the Antioxidant System in Tomato
Source: Antioxidants (Basel). 2021 Nov 30;10(12):1923. doi: 10.3390/antiox10121923 (PMC8750784; doi:10.3390/antiox10121923)
Supplement: Supplementary file 1 [file antioxidants-10-01923-s001.zip › antioxidants-1484012-supplementary.pdf]

antioxidants

Supplementary Materials: A Novel Role of Pipecolic Acid Biosynthetic Pathway in Drought Tolerance through the Antioxidant System in Tomato

Ping Wang [1], Qian Luo [1], Weicheng Yang [1], Golam Jalal Ahammed [1], Shuting Ding [1], Xingyu Chen [1], Jiao Wang [1], Xiaojian Xia [1,2] and Kai Shi [1,2,*]

[1] Department of Horticulture, Zhejiang University, Hangzhou 310058, China; 11916061@zju.edu.cn (P.W.); 11916062@zju.edu.cn (Q.L.); 3170100209@zju.edu.cn (W.Y.); ahammed@haust.edu.cn (G.J.A.); 3130100256@zju.edu.cn (S.D.); 12016053@zju.edu.cn (X.C.); 11616046@zju.edu.cn (J.W.); xiaojianxia@zju.edu.cn (X.X.)
[2] Zhejiang Provincial Key Laboratory of Horticultural Plant Integrative Biology, Hangzhou 310058, China
* Correspondence: kaishi@zju.edu.cn

Table S1. Primers used for various applications in the present study.

Genes	Accession Numbers	Forward Primer (5′–3′)	Reverse Primer (5′–3′)	Application
SlALD1	Solyc11g044840	gattgtgttctccaaacaatcccac	aaacgtgggattgtttggagaacac	CRISPR/Cas9 gene-editing vector construction
SlFMO1	Solyc07g04243	gattgtacttggctagttgtttag	aaacctaaacaactagccaagtac	CRISPR/Cas9 gene-editing vector construction
Slald1	Solyc11g044840	catttccagtgagttactct	ctttctagaacccgggattt	Mutant plants verification
Slfmo1	Solyc07g04243	cctaagaatatcgaaatttg	atggctattatccgctcgaa	Mutant plants verification
SlALD1	Solyc11g044840	cgggttctagaaaggttgcc	caatccaccagcctgagcta	qRT-PCR
SlFMO1	Solyc07g04243	cactgaggcggataagggaa	ggaactctaccagactgcga	qRT-PCR
SlALD1	Solyc11g044840	ctctcgagctttcgcgagctc<br>atgttttctctttgcatgcaacc	gcccttgctcaccatggatccattt<br>gtgtgacaaagtaaggttatgagtc	Subcellular localization
SlFMO1	Solyc07g04243	ctctcgagctttcgcgagctc<br>atggctattatccgctcgaaaa	gcccttgctcaccatggatcc<br>cttctcctctgcataatcttggc	Subcellular localization
